# FCGR3A Is a Prognostic Biomarker and Correlated with Immune Infiltrates in Lower-Grade Glioma

**DOI:** 10.1155/2022/9499317

**Published:** 2022-06-26

**Authors:** Kai Sun, Xiaowei Fei, Mingwei Xu, Ruxiang Xu, Minhui Xu

**Affiliations:** ^1^Department of Neurosurgery, Daping Hospital, Army Medical University, Chongqing 400042, China; ^2^Department of Neurosurgery, The First Affiliated Hospital of the Fourth Military Medical University, Xi'an 710032, China; ^3^Department of Neurosurgery, Sichuan Provincial People's Hospital, University of Electronic Science and Technology of China, Chengdu 610072, China

## Abstract

Low-grade gliomas (LGGs) are primary invasive brain tumors that grow slowly but are incurable and eventually develop into high malignant glioma. Fc fragment of IgG receptor IIIa (FCGR3A) gene polymorphism may correlate with some cancers' treatment responses. However, the expression and prognosis value of FCGR3A and correlation with tumor-immune infiltrate in LGG remain unclear. FCGR3A mRNA expression in gastric cancer (GC) was examined using TIMER and GEPIA databases. Correlations between FCGR3A expression and clinicopathological parameters were analyzed using ULACAN and CGGA databases. GEPIA, OncoLnc, and ULACAN databases were used to examine the clinical prognostic significance of FCGR3A in LGG. TIMER was used to analyze the correlations among FCGR3A and tumor-infiltrating immune cells. Signaling pathways related to FCGR3A expression were identified by LinkedOmics. We found that FCGR3A expression was higher in LGG than in normal tissue and was correlated with various clinical parameters. In addition, high FCGR3A expression predicted poor overall survival in LGG. More importantly, FCGR3A expression positively correlated with immune checkpoint molecules, including PD1, PD-L1, PD-L2, CTLA4, LAG-3 and TIM-3, and tumor-associated macrophage (TAM) gene markers in LGG. GO and KEGG pathway analyses indicated that TUBA1C may potentially regulate the pathogenesis of LGG through immune-related pathways. These findings indicated that FCGR3A plays a vital role in the infiltration of immune cells and could constitute a promising prognostic biomarker in LGG patients.

## 1. Introduction

Lower-grade glioma (LGG) is a diverse group of primary brain tumors, mainly including world health organization grades II and III diffuse low-grade and intermediate-grade gliomas [1]. LGG exhibits significant intrinsic heterogeneity concerning tumor biological behavior [2]. Although comprehensive treatments have been made in LGG, including neurosurgical resection, chemotherapy, and radiotherapy [3], survival has not improved significantly [4]. Recurrently mutated genes like IDH1, IDH2, TP53, and ATRX are well-recognized factors for the prognosis of patients with LGGs in clinical practice [5, 6]. Other molecular markers, including 1p/19q codeletion and MGMT promoter methylation, are important prognostic factors for LGGs [7]. However, these clinicopathological and genetic factors fail to evaluate survival outcomes accurately. Patients with the same risk factors might have conflicting results.

Consequently, a more comprehensive study is needed to increase the prognostic and predictive accuracy of the current assessment system. Numerous studies have provided evidence that cancer progression and recurrence are driven by not only the tumor's underlying genetic changes but also the tumor microenvironment (TME) [8–10]. Increasing evidence has confirmed that immune cells in the TME are involved in tumor progression and recurrence. More importantly, immune checkpoint inhibitors (ICIs) have made an indelible survival improvement for various cancers [11]. However, previous studies reported that the clinical benefit was limited in gliomas treated with PD-1 inhibitors [12, 13]. Studies indicated that combination therapy with immune checkpoint blockade might be for gliomas [14, 15]. At the same time, the immune infiltration signature has reliable prognostic and predictive value for patients with LGGs and is a potential biomarker for cotargeting immunotherapy [16]. Therefore, it is urgently needed to find novel biomarkers predicting prognosis and immune infiltrates for LGG.

Fc fragment of IgG receptor IIIa (FCGR3A, also known as CD16A) is expressed on natural killer (NK) cells as an integral membrane glycoprotein anchored through a transmembrane peptide. FCGR3A, which resides in Kupffer cells (KCs), can contribute to the inhibition of the growth of liver tumor cells [17]. More interestingly, FCGR3A gene polymorphism was associated with an increased risk of low-grade precursor lesions of cervical carcinoma [18]. FCGR3A gene polymorphism was positively associated with increased genetic risk of colorectal carcinoma [19]. The study found that FCGR3A gene polymorphisms may correlate with response to frontline rituximab plus cyclophosphamide/doxorubicin/vincristine/prednisone (R–CHOP) with diffuse large B-cell lymphoma (DLBCL) [20]. However, another research found that FCGR3A 158 V/F polymorphism was not associated with the response to frontline R–CHOP therapy in patients with DLBCL [20]. One of the accepted mechanisms of chemotherapy responded action is Fc*γ* receptor- (Fc*γ*R-) dependent antibody-dependent cellular cytotoxicity (ADCC) mediated by various immune effectors such as macrophages and natural killer cells [21]. ADCC occurs when the Fc portion of the tumor-bound antibody is recognized by the Fc*γ*Rs [22]. More importantly, analysis based on TCGA and CGGA databases identified FCGR3A as an immune‐related gene in LGG [23]. However, the expression and prognosis value of FCGR3A and correlation with tumor-immune infiltrate in LGG remain unclear.

In this study, we first analyzed the mRNA expression of FCGR3A in different cancers and then focused on LGG. We also explored the correlation between FCGR3A expression and clinical parameters in LGG. We then analyzed the impact of FCGR3A on the prognosis of LGG. In addition, we evaluated the relationship between FCGR3A and immune infiltration levels in LGG. Moreover, we examined the relationship between FCGR3A and immune checkpoint molecules in LGG. In the end, we performed the Gene Ontology and KEGG pathway enrichment analyses related to FCGR3A. These results shed light on the critical role of FCGR3A in LGG and provide an underlying mechanism between FCGR3A and tumor-immune interactions.

## 2. Materials and Methods

### 2.1. GEPIA Database Analysis

The Gene Expression Profiling Interactive Analysis (GEPIA) (https://gepia.cancer-pku.cn/index.html) [24] is a web database based on the gene expression analysis of 9,736 tumors and 8,587 healthy tissue samples from The Cancer Genome Atlas (TCGA) and The Genotype-Tissue Expression (GTEx) databases. We used the “General” tab to analyze the FCGR3A mRNA expression in 33 cancer types. We used GEPIA to generate survival curves.

We determined the OS and DFS rates of FCGR3A in LGG by the “Survival” tab. The “Correlation” tab and Spearman's method were used to determine a correlation between FCGR3A and related genes. FCGR3A values were represented on the *x*-axis, and gene values were represented on the *y*-axis.

### 2.2. Timer Database Analysis

The TIMER database (https://cistrome.shinyapps.io/timer/) [25], which includes 10,897 samples across 32 cancer types from TCGA, is a comprehensive resource for estimating the abundance of six types of infiltrating immune cells, including B cells, CD4^+^ T cells, CD8^+^ T cells, neutrophils, macrophages, and DCs. We analyzed FCGR3A expression in different cancer types using DiffExp module, and the correlation of FCGR3A expression with the abundance of immune infiltrates via the gene module. When considering tumor purity, partial correlations between variables are shown on the left-most panel of the figure or table. In addition, relationships between FCGR3A expression and publicly available gene markers of TIICs were explored using correlation modules. Spearman's method was used to determine the correlation coefficient.

### 2.3. UALCAN Database

UALCAN (https://ualcan.path.uab.edu/index.html) [26] is a portal for facilitating tumor subgroup gene expression and survival analyses. It allows the relative expression of genes between tumors and standard samples and different.

Tumor subgroups are based on the sample type, individual tumor stage, major subclasses, and other clinical-pathological features. We entered the target gene FCGR3A on the TCGA module of the website, selected brain lower-grade glioma (LGG), and obtained the differential expression of the target gene in LGG and normal tissues. This study will analyze the differential expression of FCGR3A from various angles, such as tumor grade (grade 2/grade 3), histological subtypes (astrocytoma, oligoastrocytoma, and oligodendroglioma), and TP53 mutation status (TP53-mutant/TP53-nonmutant). It also generates survival curves and determines OS rate of FCGR3A (high expression and low/medium expression) in LGG by the “Survival” tab. A *p* -value less than 0.05 is considered significant.

### 2.4. OncoLnc Database Analysis

OncoLnc (https://www.oncolnc.org/) [27] analyzed the correlation between FCGR3A expression and survival. We entered the target gene FCGR3A on the website's home page, selected brain lower-grade glioma (LGG), entered 50% both in lower and upper percentile, and obtained Kaplan plot FCGR3A in LGG. The Cox correlation coefficient and *p* -value were calculated.

### 2.5. CGGA Database Analysis

A total of mRNA microarray data from 693 samples were downloaded from CGGA (https://www.cgga.org.cn/) [28]. Only the samples in accord with the inclusion criteria were included in the analysis. The inclusion criteria were (1) patients with WHO grade II or III and (2) patients with complete clinical and transcriptional data. GraphPad Prism software was used to generate a survival curve, and the log-rank test was used to assess the statistical significance.

### 2.6. LinkedOmics Database Analysis

LinkedOmics (https://www.linkedomics.org/login.php) [29] is a publicly available portal that includes multiomics data from all 32 TCGA cancer types. The LGG sample cohort with 516 patients (platform: HiSeq RNA; analysis level: gene) was used to analyze the genes associated with FCGR3A in LGG. The GESA tool in LinkedOmics database was used to perform the Gene Ontology and KEGG pathway enrichment analyses. Spearman's method was used to determine the correlation coefficient. A *p* -value less than 0.05 is considered significant.

### 2.7. Gene Set Enrichment Analysis

Gene set enrichment analysis (GSEA) is a computational method that detects whether an a priori defined set of genes show statistically significant differential expression between high and low.

*Expression Groups.* Twenty datasets and phenotype label files were generated and uploaded into R GSEA package. The phenotype labels were FCGR3A-high and FCGR3A-low. Gene set permutations were conducted 1000 times for each analysis. Gene sets with a normal *p* < 0.05 and false discovery rate (FDR) < 0.25 were considered as enriched.

### 2.8. Genetic Alteration Analysis

After logging into the cBioPortal web (https://www.cbioportal.org/), we chose the “TCGA Pan Cancer Atlas Studies” in the “Quick Select” section and entered “FCGR3A” for queries of the genetic alteration characteristics of FCGR3A. The “Cancer Types Summary” module observed the alteration frequency, mutation type, and CNA (copy number alteration) across all TCGA tumors were observed in the “Cancer Types Summary” module. The mutated site information of FCGR3A can be displayed in the schematic diagram of the protein structure or the three-dimensional (3D) structure via the “Mutations” module.

### 2.9. Statistical Analysis

Low and high FCGR3A expression groups were established based on the median FCGR3A mRNA expression value in the separate datasets. Student's *t*-test determined the difference in continuous indexes with normal distribution between the two groups and persistent indexes with a skew distribution. Kaplan-Meier curves were utilized to evaluate the prognostic significance of FCGR3A expression. *p* -values less than 0.05 on both sides were statistically significant.

## 3. Results

### 3.1. The Expression of FCGR3A in Cancers and LGG

**Figure 1:**
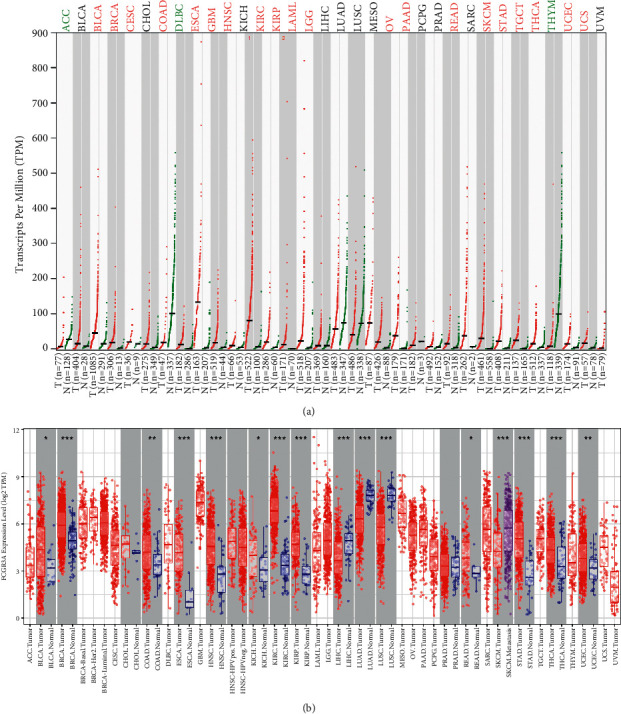
FCGR3A mRNA expression in different types of human cancers was determined with (a) GEPIA (red means that FCGR3A mRNA expression was upregulated in tumor tissues, while green was downregulated, and black was no different), (b) TIMER. ^*∗∗∗*^*p* < 0.001, ^*∗∗*^*p* < 0.01, and ^*∗*^*p* < 0.05.

We used the GEPIA database to study differences in FCGR3A expression across 33 TCGA cancer types and TCGA and GTEx normal tissues.  Figure 1(a) shows that FCGR3A expression was significantly higher in most types of cancer, including lower-grade glioma (LGG). To provide a more comprehensive evaluation of FCGR3A expression in cancers, we used TIMER database to compare FCGR3A expression in tumors and adjacent normal tissues. Similarly, FCGR3A expression was elevated in many cancers (Figure 1(b)). These results imply that FCGR3A may function as an oncogene in various cancers.

**Figure 2:**
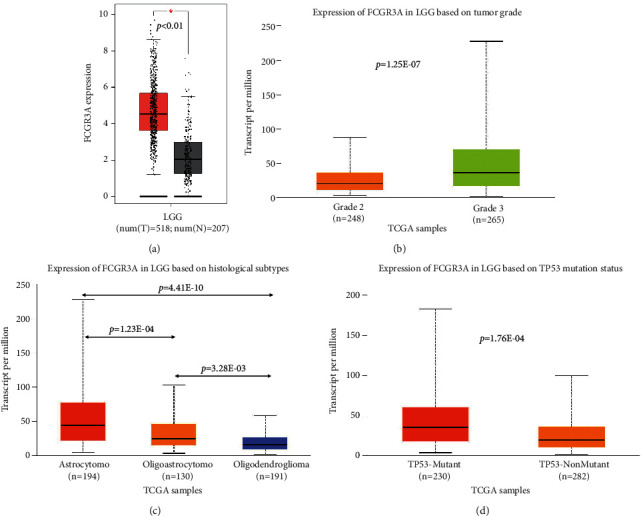
FCGR3A mRNA expression in LGG was determined with (a) GEPIA and in LGG patients with (b) different grades. (c) Histological subtypes and (d) TP53 mutation status were determined with ULACAN.

**Figure 3:**
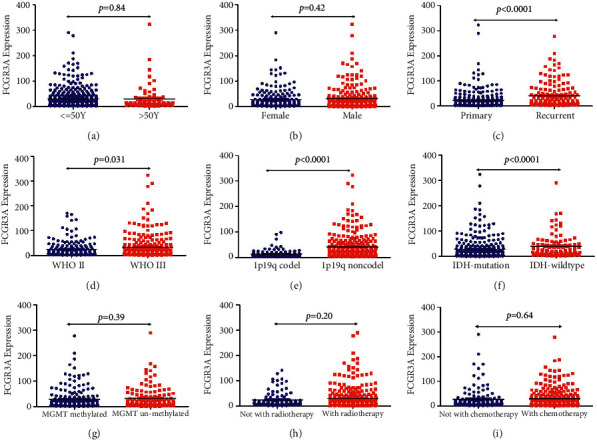
CGGA analyzed FCGR3A mRNA expression in LGG patients with different clinical parameters.

We then focused on the expression of FCGR3A in LGG. FCGR3A expression was higher in LGG than in normal tissue (Figure 2(a)). Moreover, subgroup analyses found that FCGR3A expression was significantly elevated in LGG patients with WHO grade 3, astrocytoma, and TP53 wild type (Figures 2(b)–2(d)). We further used the CGGA-LGG database to compare the difference in FCGR3A mRNA expression in groups divided by age, gender, cancer type, WHO grade, 1p19q codel, IDH1-mutation, MGMT methylated, radiotherapy treatment, and chemotherapy treatment. As clearly exhibited in Figure 3, FCGR3A mRNA expression was remarkably different in groups stratified by cancer type (recurrent patients) (*p* < 0.001), WHO grade (*p*=0.031), 1p19q codeletion (*p* < 0.001), and IDH1-mutation (*p* < 0.001), indicating the close correlation of FCGR3A mRNA expression with a series of significant clinical parameters. No association was found between FCGR3A mRNA expression and age, gender, MGMT methylated radiotherapy treatment, and chemotherapy treatment history. In addition, survival analyses found that some clinical parameters (cancer type (recurrent patients), WHO grade, 1p19q codeletion, and IDH1-mutation) were the prognosis predictors of LGG patients (Figure S1).

### 3.2. The Predictive Value of FCGR3A in Cancers and LGG

**Figure 4:**
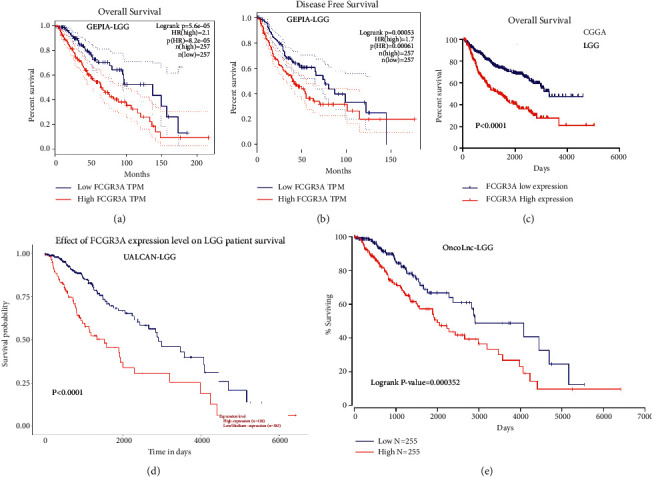
Kaplan-Meier survival curves comparing FCGR3A high and low expression in LGG. (a) OS and (b) DFS by GEPIA database; (c) OS by CGGA database; (d) OS by UALCAN database; (e) OS by OncoLnc database. DFS: disease-free survival; OS: overall survival.

We investigated the impact of FCGR3A expression on survival rates in different cancers using the GEPIA and OncoLnc databases. The relationships between FCGR3A expression and prognosis in various cancers are shown in Table S1. In the GEPIA database, high FCGR3A expression was associated with poorer overall survival (OS) in LGG (Figure 4(a)) and uveal melanoma (UVM). In contrast, it was associated with better OS in cholangiocarcinoma (CHOL) and cutaneous skin melanoma (SKCM). In addition, high FCGR3A expression was associated with poorer disease-free survival (DFS) in esophageal carcinoma (ESCA), glioblastoma multiforme (GBM), LGG (Figure 4(b)), and prostate adenocarcinoma (PRAD). In the OncoLnc database, high FCGR3A expression was associated with poorer OS in LGG (Figure 4(e)) and better OS in SKCM. Furthermore, the survival analysis using the online database UALCAN also found that high FCGR3A expression was associated with poorer OS in LGG (Figure 4(d)). Finally, we confirmed the prognostic value of FCGR3A expression in the CGGA dataset by log-rank test. LGG patients with high levels of FCGR3A mRNA experienced a much shorter OS time than LGG patients with low levels of FCGR3A mRNA (Figure 4(c)).

### 3.3. FCGR3A Expression Is Correlated with the Immune Infiltration Level in LGG

Therefore, we investigated the correlation of FCGR3A expression with immune infiltration levels in 32 cancer types from the TIMER database. The analysis showed that FCGR3A expression was associated with tumor purity in 28 cancer types and B cell infiltration levels in 24 cancer types. In addition, FCGR3A expression was associated with CD8^+^ T cell levels in 25 cancer types, CD4^+^T cell levels in 28 cancer types, macrophage levels in 27 cancer types, neutrophil levels in 30 cancer types, and dendritic cell (DC) levels in 30 cancer types (Table S2).

**Figure 5:**
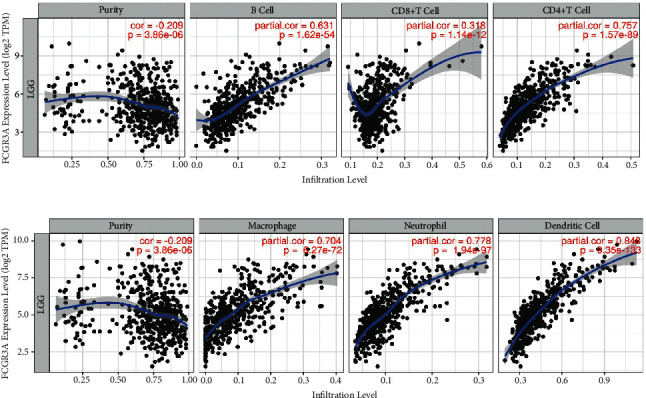
Correlation analysis between FCGR3A expression with immune infiltration level in LGG using TIMER database.

FCGR3A expression was positively correlated with the levels of infiltrating B cells, CD8^+^ T cells, CD4^+^ T cells, macrophages, neutrophils, and DCs in LGG (Figure 5). These findings strongly indicate that FCGR3A plays a vital role in immune infiltration in LGG.

### 3.4. Correlation Analysis between FCGR3A Expression and Immune Markers

To better understand the relationship between FCGR3A and various infiltrating immune cells, we analyzed the correlations between FCGR3A expression and the marker genes of different immune cells and functional T cells in LGG using the TIMER and GEPIA databases.  Table 1 shows that FCGR3A expression was associated with most marker genes of the various immune and T cells in LGG.

**Figure 6:**
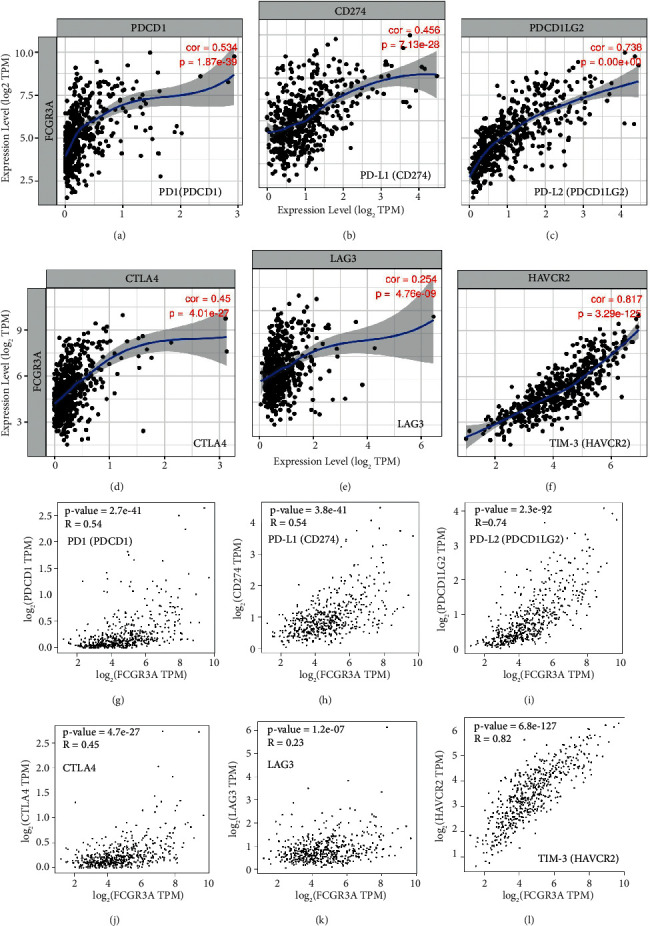
Correlation analysis between FCGR3A expression with immune checkpoint molecules in LGG using TIMER and GEPIA databases. (a) PD1 (PDCD1); (b) PD-L1 (CD274); (c) PD-L2 (PDCD1LG2); (d) CTLA4; (e) LAG3, (f) TIM-3 (HAVCR2) from TIMER; (g)PD1 (PDCD1); (h) PD-L1 (CD274); (i) PD-L2 (PDCD1LG2); (j) CTLA4; (k) LAG3; (l) TIM-3 (HAVCR2) from GEPIA.

Interestingly, FCGR3A expression was significantly associated with gene markers of B cells, monocytes, tumor-associated macrophages (TAMs), M2 macrophages, DCs, Th2 cells, and T cell exhaustion in LGG (Table 1). This analysis showed that FCGR3A expression was related to TAM-related genes and markers (Table 1). These results further reveal that FCGR3A has a strong relationship with TAM infiltration. We also found a significant relationship between FCGR3A and DC markers. In addition, we found that FCGR3A expression was significantly associated with immune checkpoint molecules, including PD1 (PDCD1) (*r* = 0.534, *p*=1.87*e* − 39), PD-L1 (CD274) (*r* = 0.456, *p*=7.13*e* − 28), PD-L2 (PDCD1LG2) (*r* = 0.738, *p*=0.00*e* − 00), CTLA4 (*r* = 0.45, *p*=4.01*e* − 27), LAG3 (*r* = 0.254, *p*=4.76*e* − 09), and TIM-3 (HAVCR2) (*r* = 0.817, *p*=3,29*e* − 125) (Figures 6(a)–6(f)). Similarly, GEPIA database analysis showed that FCGR3A expression was also significantly associated with immune checkpoint molecules in LGG (Figures 6(g)–6(l)). These results further suggest that FCGR3A plays a role in immune escape in the LGG microenvironment.

### 3.5. Enrichment Analysis of YTHDF2 Functional Networks in LGG

**Figure 7:**
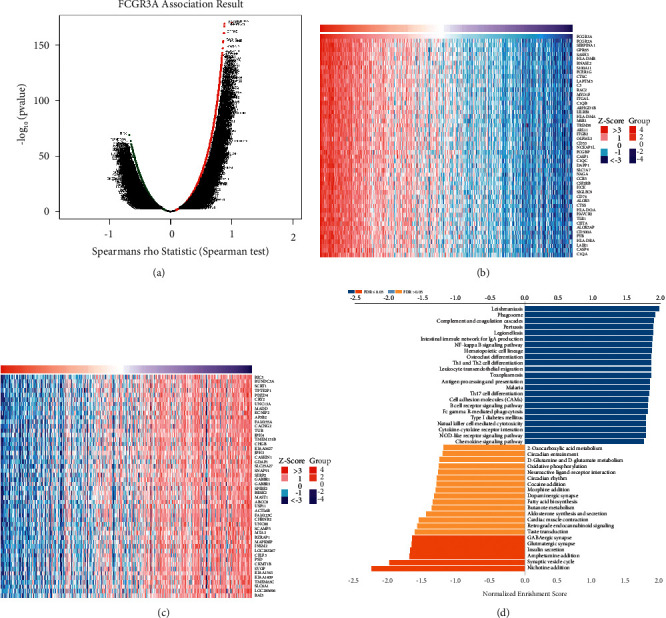
Enrichment analysis of FCGR3A functional networks in LGG by LinkedOmics. (a) Volcano plot of genes differentially expressed in correlation with FCGR3A. (b, c) Heat maps of genes positively and negatively correlated with FCGR3A (top 50). (d) KEGG pathway analysis of FCGR3A by GSEA.

We used the LinkedOmics database to analyze FCGR3A mRNA sequencing data from 516 LGG patients. The volcano plot in Figure 7(a) shows that the genes positively correlated with FCGR3A (dark-red dots) and genes negatively correlated with FCGR3A (dark-green dots) (FDR < 0.05). The 50 significant gene sets positively and negatively associated with FCGR3A are shown in the heat map (Figures 7(b) and 7(c)). The LinkedOmics GESA tool was used to perform the Gene Ontology and KEGG pathway enrichment analyses (Table S3 and Figure 7(d)). As can be seen in Table S3, the genes associated with FCGR3A were primarily found in biological processes (adaptive immune response, regulatory of immune effector process, acute inflammatory response, leukocyte cell-cell adhesion, lymphocyte activation involved in immunological reaction, and immune response-regulating signaling pathway) and cellular components (blood microparticle, secretory granule membrane, immunological synapse, and MHC protein c) (antigen binding, cytokine receptor activity, immunoglobulin binding, cytokine binding, neurotransmitter receptor activity, and neurotransmitter binding). KEGG pathway analysis shows that the genes correlated with FCGR3A were more enriched in chemokine signaling pathway, intestinal immune network for IgA production, NF-kappa B signaling pathway, Th1 and Th2 cell differentiation, Th17 cell differentiation, cell adhesion molecules (CAMs), B cell receptor signaling pathway, Fc gamma R-mediated phagocytosis, and natural killer cell-mediated cytotoxicity signaling pathway.

### 3.6. Gene Set Enrichment Analysis

**Figure 8:**
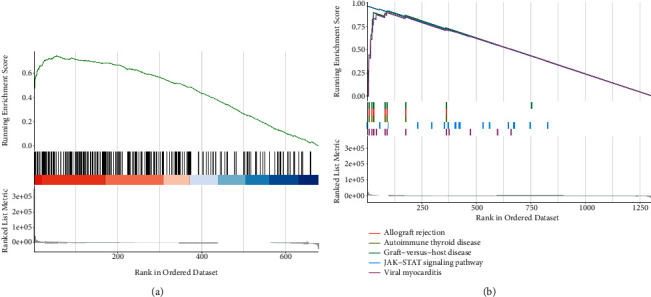
GSEA with GO term and KEGG pathway. (a) GO term analysis revealed correlated pathways. (b) KEGG pathways showed the most five correlated pathways.

To clarify the biological function of FCGR3A expression, GSEA was performed using GO terms and the KEGG pathway. The screening condition for the result was normalized enrichment score |NSE| < 1 (*p* < 0.05). Based on the absolute value of the normalized enrichment score, we selected the five most relevant signal pathways. The GO terms showed that regulation of stress response was most positively correlated with FCGR3A expression (Figure 8(a)). The KEGG pathway revealed the following five most relevant categories: allograft rejection, autoimmune thyroid disease, graft-versus-host disease, JAK−STAT signaling pathway, and viral myocarditis (Figure 8(b)). Comprehensive analysis of the previously mentioned results revealed that the FCGR3A gene promoted allograft rejection, autoimmune thyroid disease, graft-versus-host disease and JAK−STAT signaling pathway, viral myocarditis, and the stress response.

### 3.7. Genetic Alteration Analysis Data

**Figure 9:**
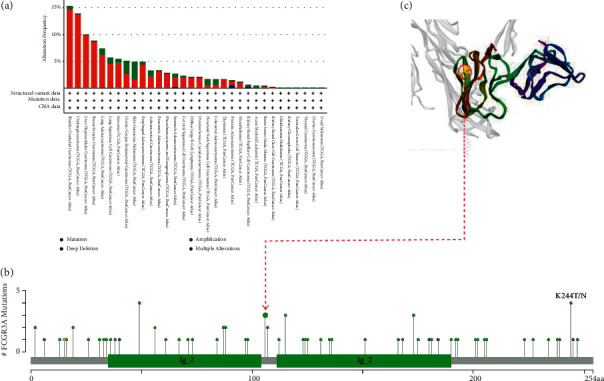
Mutation feature of FCGR3A in different tumors of TCGA. We analyzed the mutation features of FCGR3A for the TCGA tumors using the cBioPortal tool. The alteration frequency with mutation type (a) and mutation site (b) are displayed. We display the mutation site with the I142Mfs^*∗*^21 in the 3D structure of FCGR3A (c).

We observed the genetic alteration status of FCGR3A in different tumor samples of the TCGA cohorts. As shown in Figure 9(a), the highest alteration frequency of FCGR3A (>3.8%) appears for patients with skin cutaneous melanoma with “mutation” as the primary type. The “amplification” type of CNA was the primary type in ovarian cancer cases, which showed an alteration frequency of ∼5% (Figure 9(a)). It is worth noting that all cholangiocarcinoma cases, liver hepatocellular carcinoma cases, pancreatic adenocarcinoma cases, pheochromocytoma, paraganglioma cases, and diffuse large B-cell lymphoma with genetic alteration (>2% frequency) had copy number amplification of FCGR3A (Figure 9(a)). The types, sites, and case numbers of the FCGR3A genetic alteration are further presented in Figure 9(b). We found that missense mutation of FCGR3A was the primary type of gene alteration and I142Mfs^*∗*^21 alteration between the lg_2 domain, which was detected in one case of cervical squamous cell carcinoma, one case of lung squamous cell carcinoma, one case of uterine endometrioid carcinoma (Figure 9(b)), can induce a frameshift mutation of the FCGR3A gene, translation from I (isoleucine) to M (methionine) at the 142 sites of FCGR3A protein, and the subsequent FCGR3A protein truncation.

## 4. Discussion

This is the first study to comprehensively analyze the expression and prognostic value of FCGR3A in LGG. We found that FCGR3A expression was higher in LGG compared with standard samples. Moreover, FCGR3A mRNA expression was higher in LGG patients with 1p19q nonmodel and IDH1-wildtype, which were the poor prognosis predictors of LGG. More importantly, high FCGR3A expression was also correlated with more deficient survival in patients with LGG. These results implied that FCGR3A was a prognostic factor in LGG.

In the present study, FCGR3A expression was positively correlated with the levels of infiltrating B cells, CD8^+^ T cells, CD4^+^ T cells, macrophages, neutrophils, and DCs in LGG. Notably, FCGR3A expression was associated with TAM and M2 macrophage markers, including CSF1R, IL-10, TGF*β* (TGFB1), PD-L1 (CD274), PD-L2 (PDCD1LG2), CD68, CD80, CD86, CD163, VSIG4, and MFG-E8. Cancer patients' tumor immunosuppressive microenvironment plays a crucial role in regulating the growth and spread of TAMs, making them promising therapeutic targets [30]. Glioma cancer stem cells (gCSCs) induced the secretion of the immunosuppressive cytokine interleukin-10 (IL-10) and transforming growth factor (TGF*β*1) and IL-10 and facilitated immunosuppression [31]. Cancer-associated fibroblasts- (CAF-) educated cells inhibited T cell proliferation through the production of TGF*β* and IL-10 and facilitated an immunosuppressive microenvironment [32]. Furthermore, inhibiting the colony-stimulating factor-1 receptor (CSF1R) in TAMs might significantly reduce tumor-initiating cells (TICs), hence relieving immunosuppression and overcoming TIC-mediated chemotherapeutic resistance [33]. High expression CD163 (mainly expressed in M2 macrophages) was associated with poorer survival in LGG patients [34]. VSIG4 can inhibit CD4^+^ and CD8^+^ T-cell proliferation. VSIG4 induced epithelial-mesenchymal transition (EMT) and significantly promoted invasion and migration in glioblastoma U-87 MG cells [35]. VSIG4 is highly expressed and correlated with the poor prognosis of high-grade glioma patients [36]. Milk-fat globule-epidermal growth factor-VIII (MFG-E8) regulates the immunogenicity of DC [37]. MFG-E8 is important for embryonic stem cell-mediated T cell immunomodulation [38]. MFG-E8 (the downstream factor of TAMs) promoted tumorigenicity and anticancer drug resistance in cancer stem/initiating cells (CSCs) mainly by activating the signal transducer and activator of transcription-3 (STAT3) and sonic hedgehog pathways [39]. Therefore, we hypothesize that FCGR3A may promote the immunosuppressive thought regulation of TAMs.

In addition, FCGR3A expression was correlated with DCs markers. DCs can promote tumor metastasis by increasing Treg cells and reducing CD8^+^T cell cytotoxicity [40]. More importantly, we found that FCGR3A expression was significantly associated with immune checkpoint molecules (PD1, PD-L1, PD-L2, CTLA4, LAG3, and TIM-3), some of which were highly expressed in patients in the high-risk group of LGG [41]. PD-1 promoter methylation is a prognostic factor in LGG patients with IDH-mutated [42]. Higher PD-L1 expression was found in IDH-wild typed LGG than in IDH-mutated cases [43]. Similarly, PD-L2 expression was upregulated in higher grade glioma and IDH-wild-type glioma [44]. High PD-L2 expression was associated with poor survival in GBM. In addition, high PD-L1 and PD-L2 expression were also found to be associated with poor survival in LGG, respectively [44]. Higher CTLA-4 expression was associated with more inferior OS in patients with LGG based on TCGA and CGGA databases [45]. TIM-3 is a crucial T cell exhaustion regulator [46] and regulates CD103 + dendritic cells [47]. TIM-3 plays a specific role in T cell tumor-immune response in glioma [48]. High TIM-3 expression was an independent indicator of poor prognosis of glioma [48]. Therefore, TIM-3 may be a promising target when glioma gains resistance to antibodies of PD-1/PD-L1. LAG-3 ensured immune homeostasis by suppressing T cell activation and cytokines secretion. While targeting LAG-3, immunotherapy may effectively fight PD-1 resistance [49, 50]. However, previous studies reported the clinical benefit was limited in gliomas treated with PD-1 inhibitors [49, 50]. Gliomas are known to respond well to immunotherapy treatments such as DC vaccinations, peptide immunotherapy, and CAR-T cells as well as oncolytic viruses [51, 52]. PD-1 and TIGIT dual checkpoint blockade included antitumor immunity and survival in a murine GBM model [53]. Blocking PD-1/PD-L1 interactions combined with MLN4924 therapy is a potential treatment for glioma patients [54]. Gliomas treated with a tripartite regimen (DC vaccine, PD-1 monoclonal antibody, and colony-stimulating factor 1 receptor inhibitor (PLX3397)) had included survival in vivo [55]. Studies indicated that combination therapy with immune checkpoint blockade is effective for gliomas [14, 15]. Therefore, targeting FCGR3A with PD1 immunotherapy may effectively fight PD-1 resistance in LGG.

GSEA enrichment analysis evaluates the distribution trend of genes in a predefined gene set in a gene table ranked by their relevance to phenotype, thereby judging their contribution to phenotype. The results of GSEA analysis indicated that LGG samples in the high expression group of FCGR3A gene were mainly enriched in allograft rejection, autoimmune thyroid disease, graft-versus-host disease, JAK−STAT signaling pathway, viral myocarditis, and the response of stress signaling pathway, indicating that FCGR3A may be involved in multiple biological processes in the occurrence and development of LGG. Mutation analysis of the FCGR3A gene using the cBioportal online website found that LGG patients had gene mutations, and the mutation type was copy number amplification. FCGR3A has copy number amplification in multiple cancers, which may have no effect or benefit on the expression products. Still, most may lead to deleterious or lethal consequences, and this mutation may serve as a new potential biomarker for developing new cancer treatment strategies.

In addition, the enrichment analyses showed that FCGR3A influences the tumor development process through multiple immune-related pathways, including the chemokine signaling pathway, intestinal immune network for IgA production, NF-kappa B signaling pathway, Th1 and Th2 cell differentiation, Th17 cell differentiation, cell adhesion molecules (CAMs), B cell receptor signaling pathway, Fc gamma R-mediated phagocytosis, and natural killer cell-mediated cytotoxicity signaling pathway. These results indicated that FCGR3A was involved in immune-related ways in the TME of LGG. However, because this bioinformatics analysis was performed based on TCGA or GEO datasets, further biological experiments are needed to validate future results.

We knew FCGR3A was involved in a variety of biological processes, molecular functions, and cellular components when we looked beside him, such as FC receptor signaling pathway, cell surface receptor signaling pathway for immune response regulation, phosphotyrosine residue binding, immunoglobulin binding, plasma membrane, and whole membrane. KEGG analysis is used to identify essential signal pathways. Fc *γ* R-mediated phagocytosis and osteoclast differentiation are of prime importance among its related pathways. Based on the PPI network and correlation scores produced by Guo and Xu [56], FCGR3A, SYK, and HCK were the most relevant neighboring genes. FCGR3A (low-affinity immunoglobulin *γ* Fc region receptor III-A) is the Fc region receptor for IgG and binds to either pooled or aggregated IgG and monomeric IgG. It primarily mediates ADCC and other antibody-dependent responses [57]. SYK is a nonreceptor tyrosine kinase that mediates signal transduction downstream of transmembrane receptors, including classical immune receptors [58]. HCK is present in hematopoietic cells, and it transmits signals from cell surface receptors and regulates the innate immune response. It also acts downstream of receptors that bind to the Fc region of immunoglobulins, such as FCGR1A, FCGR2A, and CSF3R [59]. All three genes are associated with various cancer types or drug responses [60–62]. In fact, their PPI network proves a highly significant positive correlation between FCGR3A and FCGR1A. The existing evidence that FCG1A is positively correlated with immune infiltration levels of various cancers, especially cervical cancer (CESC), cholangiocarcinoma (CHOL), renal clear cell carcinoma (KIRC), and skin melanoma may help us to infer the correlation between FCGR3A and cancer.

We performed the first comprehensive bioinformatics analysis of FCGR3A expression and prognostic value in human cancers. High expression of FCGR3A correlates with poor prognosis and increased immune infiltration levels (including infiltration of B cells, CD8^+^ T cells, CD4^+^ T cells, macrophages, neutrophils, and DCs) in LGG. FCGR3A expression is significantly associated with the expression of TAM gene markers in LGG. In addition, FCGR3A expression positively correlated with immune checkpoint molecules, including PD1, PD-L1, PD-L2, CTLA4, LAG3, and TIM-3. These findings indicated that FCGR3A is essential in infiltrating immune cells and could be a promising prognostic biomarker in LGG patients.

## Figures and Tables

**Table 1:** Correlation analysis between FCGR3A and related genes and markers of immune cells in LGG using TIMER and GEPIA databases.

Description	Gene markers	LGG (TIMER)				LGG (GEPIA)	
		None		Purity			
		cor	*p*	cor	*p*	R	*p*
CD8^+^T cell	CD8A	0.2719	^*∗∗∗*^	0.2843	^*∗∗∗*^	0.3	^*∗∗∗*^
	CD8B	0.2471	^*∗∗∗*^	0.2713	^*∗∗∗*^	0.26	^*∗∗∗*^
T cell (general)	CD3D	0.5282	^*∗∗∗*^	0.5476	^*∗∗∗*^	0.49	^*∗∗∗*^
	CD3E	0.5893	^*∗∗∗*^	0.6117	^*∗∗∗*^	0.6	^*∗∗∗*^
	CD2	0.5923	^*∗∗∗*^	0.6058	^*∗∗∗*^	0.61	^*∗∗∗*^
B cell	CD19	0.4395	^*∗∗∗*^	0.4341	^*∗∗∗*^	0.55	^*∗∗∗*^
	CD79A	0.3709	^*∗∗∗*^	0.3921	^*∗∗∗*^	0.38	^*∗∗∗*^
Monocyte	CD86	0.7949	^*∗∗∗*^	0.8023	^*∗∗∗*^	0.8	^*∗∗∗*^
	CSF1R	0.6704	^*∗∗∗*^	0.6720	^*∗∗∗*^	0.67	^*∗∗∗*^
TAM	CCL2	0.5687	^*∗∗∗*^	0.5589	^*∗∗∗*^	0.58	^*∗∗∗*^
	CD68	0.7838	^*∗∗∗*^	0.7931	^*∗∗∗*^	0.78	^*∗∗∗*^
	PD-L1 (CD274)	0.4561	^*∗∗∗*^	0.4616	^*∗∗∗*^	0.54	^*∗∗∗*^
	PD-L2 (PDCD1LG2)	0.7379	^*∗∗∗*^	0.7469	^*∗∗∗*^	0.74	^*∗∗∗*^
	IL10	0.6571	^*∗∗∗*^	0.6514	^*∗∗∗*^	0.65	^*∗∗∗*^
M1 macrophage	INOS (NOS2)	−0.1623	^*∗∗∗*^	−0.1449	^*∗∗*^	−0.15	^*∗∗∗*^
	IRF5	0.7079	^*∗∗∗*^	0.7169	^*∗∗∗*^	0.7	^*∗∗∗*^
	COX2 (PTGS2)	0.1645	^*∗∗∗*^	0.1566	^*∗∗∗*^	0.21	^*∗∗∗*^
M2 macrophage	CD163	0.5708	^*∗∗∗*^	0.5581	^*∗∗∗*^	0.56	^*∗∗∗*^
	VSIG4	0.7058	^*∗∗∗*^	0.7030	^*∗∗∗*^	0.71	^*∗∗∗*^
	MS4A4A	0.6396	^*∗∗∗*^	0.6444	^*∗∗∗*^	0.65	^*∗∗∗*^
Neutrophils	CD66b (CEACAM8)	0.0304	0.4902	0.0232	0.6131	0.03	0.5000
	CD11b (ITGAM)	0.7129	^*∗∗∗*^	0.7152	^*∗∗∗*^	0.71	^*∗∗∗*^
	CCR7	0.4100	^*∗∗∗*^	0.4265	^*∗∗∗*^	0.42	^*∗∗∗*^
Natural killer cell	KIR2DL1	0.0730	0.0977	0.0914	^*∗*^	0.14	^*∗∗*^
	KIR2DL3	0.2595	^*∗∗∗*^	0.2808	^*∗∗∗*^	0.26	^*∗∗∗*^
	KIR2DL4	0.4566	^*∗∗∗*^	0.4752	^*∗∗∗*^	0.49	^*∗∗∗*^
	KIR3DL1	0.0670	0.1284	0.0715	0.1182	0.15	^*∗∗∗*^
	KIR3DL2	0.1890	^*∗∗∗*^	0.2042	^*∗∗∗*^	0.2	^*∗∗∗*^
	KIR3DL3	0.0562	0.2023	0.0362	0.4287	0.057	0.1900
	KIR2DS4	0.2158	^*∗∗∗*^	0.2379	^*∗∗∗*^	0.22	^*∗∗∗*^
Dendritic cell	HLA-DPB1	0.7614	^*∗∗∗*^	0.7645	^*∗∗∗*^	0.77	^*∗∗∗*^
	HLA-DQB1	0.6223	^*∗∗∗*^	0.6245	^*∗∗∗*^	0.51	^*∗∗∗*^
	HLA-DRA	0.8060	^*∗∗∗*^	0.8044	^*∗∗∗*^	0.81	^*∗∗∗*^
	HLA-DPA1	0.7780	^*∗∗∗*^	0.7813	^*∗∗∗*^	0.78	^*∗∗∗*^
	BDCA-1 (CD1C)	0.4226	^*∗∗∗*^	0.4342	^*∗∗∗*^	0.45	^*∗∗∗*^
	BDCA-4 (NRP1)	0.3271	^*∗∗∗*^	0.3277	^*∗∗∗*^	0.32	^*∗∗∗*^
	CD11c (ITGAX)	0.5944	^*∗∗∗*^	0.6012	^*∗∗∗*^	0.57	^*∗∗∗*^
Th1	T-bet (TBX21)	0.3742	^*∗∗∗*^	0.3753	^*∗∗∗*^	0.41	^*∗∗∗*^
	STAT4	−0.1408	^*∗∗*^	−0.1228	^*∗∗*^	−0.098	^*∗*^
	STAT1	0.5176	^*∗∗∗*^	0.5159	^*∗∗∗*^	0.54	^*∗∗∗*^
	IFN-*γ* (IFNG)	0.2490	^*∗∗∗*^	0.2547	^*∗∗∗*^	0.28	^*∗∗∗*^
	TNF-*α* (TNF)	0.2990	^*∗∗∗*^	0.2856	^*∗∗∗*^	0.32	^*∗∗∗*^
Th2	GATA3	0.4229	^*∗∗∗*^	0.4314	^*∗∗∗*^	0.4	^*∗∗∗*^
	STAT6	0.4826	^*∗∗∗*^	0.5005	^*∗∗∗*^	0.5	^*∗∗∗*^
	STAT5A	0.6670	^*∗∗∗*^	0.6630	^*∗∗∗*^	0.67	^*∗∗∗*^
	IL13	−0.0299	0.4986	−0.0314	0.4923	0.0039	0.93
Tfh	BCL6	0.1152	^*∗∗*^	0.0834	0.0681	0.1500	^*∗∗∗*^
	IL21	0.1336	^*∗∗*^	0.1174	^*∗*^	0.21	^*∗∗∗*^
Th17	STAT3	0.5832	^*∗∗∗*^	0.5700	^*∗∗∗*^	0.57	^*∗∗∗*^
	IL17A	0.0141	0.7500	−0.0082	0.8571	0.0200	0.6500
Treg	FOXP3	−0.1947	^*∗∗∗*^	−0.1748	^*∗∗∗*^	−0.14	^*∗∗*^
	CCR8	0.1704	^*∗∗∗*^	0.1853	^*∗∗∗*^	0.21	^*∗∗∗*^
	STAT5B	−0.0002	0.9962	−0.0272	0.5532	0.038	0.38
	TGF*β* (TGFB1)	0.6535	^*∗∗∗*^	0.6578	^*∗∗∗*^	0.66	^*∗∗∗*^
T cell exhaustion	PD-1 (PDCD1)	0.5345	^*∗∗∗*^	0.5401	^*∗∗∗*^	0.54	^*∗∗∗*^
	CTLA4	0.4502	^*∗∗∗*^	0.4482	^*∗∗∗*^	0.45	^*∗∗∗*^
	LAG3	0.2541	^*∗∗∗*^	0.2590	^*∗∗∗*^	0.23	^*∗∗∗*^
	TIM-3 (HAVCR2)	0.8174	^*∗∗∗*^	0.8231	^*∗∗∗*^	0.82	^*∗∗∗*^
	GZMB	0.3520	^*∗∗∗*^	0.3627	^*∗∗∗*^	0.36	^*∗∗∗*^

LGG: brain lower grade glioma; TAM: tumor-associated macrophage; Th: *T* helper cell; Tfh: follicular helper T cell; Treg: regulatory T cell; Cor: R-value of Spearman's correlation; none: correlation without adjustment. Purity: correlation adjusted by purity.*p*-value significant codes: 0 ≤ ^*∗∗∗*^ <0.001 ≤ ^*∗∗*^ <0.01 ≤ ^*∗*^ <0.05 ≤.

## Data Availability

The data used to support the findings of this study are available from the corresponding author upon request.

## Supplementary Materials

Supplementary Materials Figure S1: survival analysis of prognostic predictors in LGG patients. Table S1: prognostic values of FCGR3A in cancers analyzed by GEPIA and OncoLnc databases. Table S2: correlation analysis between FCGR3A and immune infiltration level in cancers using TIMER database. Table S3. The enrichment analysis of FCGR3A in LGG by GSEA tool of LinkedOmics database.
