# Supplementary material for: FCGR3A Is a Prognostic Biomarker and Correlated with Immune Infiltrates in Lower-Grade Glioma
Source: J Oncol. 2022 Jun 26;2022:9499317. doi: 10.1155/2022/9499317 (PMC11401682; doi:10.1155/2022/9499317)
Supplement: Supplementary Materials — Figure S1: survival analysis of prognostic predictors in LGG patients. Table S1: prognostic values of FCGR3A in cancers analyzed by GEPIA and OncoLnc databases. Table S2: correlation analysis between FCGR3A and immune infiltration level in cancers using TIMER database. Table S3. The enrichment analysis of FCGR3A in LGG by GSEA tool of LinkedOmics database. [file 9499317.f1.docx]

**Table S1. Prognostic values of FCGR3A in cancers analyzed by GEPIA and OncoLnc databases.**

| GEPIA | | | | | OncoLnc | | |
| --- | --- | --- | --- | --- | --- | --- | --- |
|  | OS | | RFS | |  | OS | |
| Cancer | HR | P-value | HR | P-value | Cancer | Cox Coefficient | P-value |
| ACC | 1.2 | 0.59 | 0.83 | 0.6 | BLCA | 0.061 | 0.43 |
| BLCA | 1.2 | 0.22 | 1.1 | 0.76 | BRCA | 0.029 | 0.74 |
| BRCA | 1.2 | 0.17 | 0.96 | 0.84 | CESC | -0.088 | 0.49 |
| CESC | 0.79 | 0.31 | 0.95 | 0.86 | COAD | 0.043 | 0.67 |
| CHOL | 0.34 | * | 0.53 | 0.18 | ESCA | 0.151 | 0.26 |
| COAD | 1.1 | 0.74 | 1 | 0.97 | GBM | 0.07 | 0.42 |
| DLBC | 0.6 | 0.48 | 1.8 | 0.35 | HNSC | -0.011 | 0.88 |
| ESCA | 1.4 | 0.12 | 1.7 | * | KIRC | 0.011 | 0.89 |
| GBM | 1.1 | 0.54 | 1.6 | * | KIRP | 0.167 | 0.26 |
| HNSC | 1.1 | 0.6 | 0.87 | 0.42 | LAML | 0.037 | 0.73 |
| KICH | 1 | 0.96 | 1.4 | 0.58 | LGG | 0.401 | *** |
| KIRC | 1.2 | 0.26 | 1.1 | 0.5 | LIHC | 0.034 | 0.73 |
| KIRP | 1.7 | 0.068 | 1.5 | 0.17 | LUAD | -0.031 | 0.66 |
| LAML | 1.1 | 0.64 | 1 | 1 | LUSC | 0.117 | 0.094 |
| LGG | 2.1 | *** | 1.7 | *** | OV | 0.038 | 0.62 |
| LIHC | 1.3 | 0.17 | 1.1 | 0.41 | PAAD | 0.097 | 0.36 |
| LUAD | 1 | 0.87 | 0.92 | 0.57 | READ | 0.297 | 0.19 |
| LUSC | 1 | 0.062 | 1.2 | 0.41 | SARC | -0.097 | 0.36 |
| MESO | 1 | 0.93 | 1 | 0.83 | SKCM | -0.346 | *** |
| OV | 0.9 | 0.37 | 0.79 | 0.06 | STAD | 0.057 | 0.54 |
| PAAD | 1.1 | 0.73 | 1.2 | 0.54 | UCEC | -0.002 | 0.98 |
| PCPG | 0.22 | 0.14 | 1.2 | 0.76 |  |  |  |
| PRAD | 0.56 | 0.41 | 1.8 | ** |  |  |  |
| READ | 2 | 0.16 | 1.4 | 0.5 |  |  |  |
| SARC | 0.84 | 0.38 | 1.1 | 0.6 |  |  |  |
| SKCM | 0.59 | *** | 0.81 | 0.075 |  |  |  |
| STAD | 1.2 | 0.36 | 0.92 | 0.68 |  |  |  |
| TGCT | 2.9 | 0.33 | 0.91 | 0.79 |  |  |  |
| THCA | 0.46 | 0.14 | 1.2 | 0.57 |  |  |  |
| THYM | 1.8 | 0.43 | 0.83 | 1.1 |  |  |  |
| UCEC | 0.78 | 0.48 | 0.8 | 0.5 |  |  |  |
| UCS | 0.72 | 0.34 | 0.54 | 0.094 |  |  |  |
| UVM | 2.8 | * | 2 | 0.13 |  |  |  |

Note: OS, overall survival; RFS, Disease Free Survival; ACC,Adrenocortical carcinoma; BLCA,Bladder Urothelial Carcinoma; BRCA,Breast invasive carcinoma; CESC,Cervical squamous cell carcinoma and endocervical adenocarcinoma; CHOL,Cholangio carcinoma; COAD,Colon adenocarcinoma; DLBC Lymphoid Neoplasm Diffuse Large B-cell Lymphoma; ESCA,Esophageal carcinoma; GBM,Glioblastoma multiforme; HNSC,Head and Neck squamous cell carcinoma; KICH,Kidney Chromophobe; KIRC,Kidney renal clear cell carcinoma; KIRP, Kidney renal papillary cell carcinoma; LAML,Acute Myeloid Leukemia; LGG,Brain Lower Grade Glioma; LIHC,Liver hepatocellular carcinoma; LUAD,Lung adenocarcinoma; LUSC,Lung squamous cell carcinoma; MESO,Mesothelioma; OV,Ovarian serous cystadenocarcinoma; PAAD,Pancreatic adenocarcinoma; PCPG,Pheochromocytoma and Paraganglioma; PRAD,Prostate adenocarcinoma; READ,Rectum adenocarcinoma; SARC,Sarcoma; SKCM,Skin Cutaneous Melanoma; STAD,Stomach adenocarcinoma; TGCT,Testicular Germ Cell Tumors; THCA,Thyroid carcinoma; THYM,Thymoma; UCEC,Uterine Corpus Endometrial Carcinoma; UCS,Uterine Carcinosarcoma; UVM,Uveal Melanoma. P-value Significant Codes: 0 ≤ *** < 0.001 ≤ ** < 0.01 ≤ * < 0.05.

**Table S2. Correlation analysis between FXGR3A and immune infiltration level in cancers in TIMER.**

|  | Purity | | B Cell | | CD8+ T Cell | | CD4+ T Cell | | Macrophage | | Neutrophil | | Dendritic Cell | |
| --- | --- | --- | --- | --- | --- | --- | --- | --- | --- | --- | --- | --- | --- | --- |
| **Cancer** | cor | p | cor | p | cor | p | cor | p | cor | p | cor | p | cor | p |
| ACC | -0.503 | *** | 0.462 | *** | 0.207 | 0.079 | 0.365 | ** | 0.592 | *** | 0.560 | *** | 0.450 | *** |
| BLCA | -0.613 | *** | -0.076 | 0.149 | 0.376 | *** | 0.313 | *** | 0.374 | *** | 0.638 | *** | 0.644 | *** |
| BRCA | -0.254 | *** | 0.423 | *** | 0.450 | *** | 0.388 | *** | 0.566 | *** | 0.661 | *** | 0.655 | *** |
| CESC | -0.310 | *** | 0.166 | ** | 0.432 | *** | 0.405 | *** | 0.356 | *** | 0.597 | *** | 0.717 | *** |
| CHOL | -0.313 | 0.063 | 0.343 | * | 0.352 | * | 0.371 | * | 0.729 | *** | 0.803 | *** | 0.496 | ** |
| COAD | -0.364 | *** | 0.200 | *** | 0.447 | *** | 0.326 | *** | 0.662 | *** | 0.746 | *** | 0.727 | *** |
| DLBC | -0.129 | 0.417 | 0.283 | 0.255 | 0.209 | 0.362 | 0.131 | 0.571 | -0.184 | 0.425 | 0.552 | ** | 0.255 | 0.264 |
| ESCA | -0.266 | *** | 0.160 | * | 0.047 | 0.531 | 0.280 | *** | 0.674 | *** | 0.269 | *** | 0.329 | *** |
| GBM | -0.553 | *** | 0.130 | 0.147 | -0.468 | *** | 0.306 | *** | 0.080 | 0.364 | 0.327 | *** | 0.472 | *** |
| HNSC | -0.282 | *** | 0.249 | *** | 0.524 | *** | 0.567 | *** | 0.768 | *** | 0.755 | *** | 0.804 | *** |
| KICH | -0.515 | *** | 0.546 | *** | 0.197 | 0.116 | 0.269 | * | 0.432 | *** | 0.147 | 0.241 | 0.765 | *** |
| KIRC | -0.297 | *** | 0.503 | *** | 0.500 | *** | 0.314 | *** | 0.639 | *** | 0.731 | *** | 0.738 | *** |
| KIRP | -0.229 | *** | 0.548 | *** | 0.527 | *** | 0.375 | *** | 0.435 | *** | 0.537 | *** | 0.727 | *** |
| LGG | -0.209 | *** | 0.631 | *** | 0.318 | *** | 0.757 | *** | 0.704 | *** | 0.778 | *** | 0.848 | *** |
| LIHC | -0.366 | *** | 0.502 | *** | 0.588 | *** | 0.260 | *** | 0.587 | *** | 0.603 | *** | 0.661 | *** |
| LUAD | -0.387 | *** | 0.171 | *** | 0.438 | *** | 0.272 | *** | 0.618 | *** | 0.719 | *** | 0.713 | *** |
| LUSC | -0.370 | *** | 0.330 | *** | 0.609 | *** | 0.288 | *** | 0.626 | *** | 0.608 | *** | 0.802 | *** |
| MESO | -0.215 | * | 0.307 | ** | 0.031 | 0.777 | 0.331 | ** | 0.339 | ** | 0.277 | * | 0.575 | *** |
| OV | -0.562 | *** | 0.243 | *** | 0.483 | *** | 0.301 | *** | 0.460 | *** | 0.636 | *** | 0.573 | *** |
| PAAD | -0.392 | *** | 0.190 | * | 0.433 | *** | 0.153 | * | 0.675 | *** | 0.656 | *** | 0.738 | *** |
| PCPG | -0.330 | *** | 0.139 | 0.073 | -0.045 | 0.560 | 0.370 | *** | 0.531 | *** | 0.242 | ** | 0.462 | *** |
| PRAD | -0.233 | *** | 0.560 | *** | 0.421 | *** | 0.503 | *** | 0.546 | *** | 0.574 | *** | 0.662 | *** |
| READ | -0.365 | *** | 0.153 | 0.072 | 0.257 | ** | 0.260 | ** | 0.406 | *** | 0.450 | *** | 0.738 | *** |
| SARC | -0.542 | *** | 0.461 | *** | 0.382 | *** | 0.393 | *** | 0.602 | *** | 0.454 | *** | 0.748 | *** |
| SKCM | -0.456 | *** | 0.235 | *** | 0.600 | *** | 0.288 | *** | 0.543 | *** | 0.738 | *** | 0.696 | *** |
| STAD | -0.191 | *** | -0.436 | *** | 0.534 | *** | 0.089 | 0.091 | 0.462 | *** | 0.681 | *** | 0.740 | *** |
| TGCT | -0.301 | *** | 0.041 | 0.618 | 0.192 | * | -0.029 | 0.724 | 0.371 | *** | 0.300 | *** | 0.368 | *** |
| THCA | -0.085 | 0.059 | 0.438 | *** | 0.410 | *** | 0.201 | *** | 0.456 | *** | 0.601 | *** | 0.723 | *** |
| THYM | 0.220 | * | -0.209 | * | -0.279 | ** | -0.452 | *** | 0.026 | 0.784 | 0.401 | *** | -0.324 | *** |
| UCEC | -0.214 | *** | 0.487 | *** | 0.408 | *** | 0.317 | *** | 0.412 | *** | 0.541 | *** | 0.583 | *** |
| UCS | -0.372 | ** | 0.236 | 0.089 | 0.088 | 0.532 | 0.521 | *** | 0.255 | 0.065 | 0.554 | *** | 0.883 | *** |
| UVM | 0.128 | 0.263 | -0.017 | 0.882 | 0.322 | ** | -0.115 | 0.321 | -0.002 | 0.987 | -0.109 | 0.348 | -0.031 | 0.795 |

ACC,Adrenocortical carcinoma; BLCA,Bladder Urothelial Carcinoma; BRCA,Breast invasive carcinoma; CESC,Cervical squamous cell carcinoma and endocervical adenocarcinoma; CHOL,Cholangio carcinoma; COAD,Colon adenocarcinoma; DLBC Lymphoid Neoplasm Diffuse Large B-cell Lymphoma; ESCA,Esophageal carcinoma; GBM,Glioblastoma multiforme; HNSC,Head and Neck squamous cell carcinoma; KICH,Kidney Chromophobe; KIRC,Kidney renal clear cell carcinoma; KIRP, Kidney renal papillary cell carcinoma; LAML,Acute Myeloid Leukemia; LGG,Brain Lower Grade Glioma; LIHC,Liver hepatocellular carcinoma; LUAD,Lung adenocarcinoma; LUSC,Lung squamous cell carcinoma; MESO,Mesothelioma; OV,Ovarian serous cystadenocarcinoma; PAAD,Pancreatic adenocarcinoma; PCPG,Pheochromocytoma and Paraganglioma; PRAD,Prostate adenocarcinoma; READ,Rectum adenocarcinoma; SARC,Sarcoma; SKCM,Skin Cutaneous Melanoma; STAD,Stomach adenocarcinoma; TGCT,Testicular Germ Cell Tumors; THCA,Thyroid carcinoma; THYM,Thymoma; UCEC,Uterine Corpus Endometrial Carcinoma; UCS,Uterine Carcinosarcoma; UVM,Uveal Melanoma. P-value Significant Codes: 0 ≤ *** < 0.001 ≤ ** < 0.01 ≤ * < 0.05.

**Table S3. The enrichment analysis of FCGR3A in LGG by GSEA tool of LinkedOmics database.**

|  | **Gene Set** | **Description** | **Size** | **P-Value** |
| --- | --- | --- | --- | --- |
| **BP** | GO:0002250 | adaptive immune response | 368 | 0 |
|  | GO:0002697 | regulation of immune effector process | 365 | 0 |
|  | GO:0002526 | acute inflammatory response | 151 | 0 |
|  | GO:0007159 | leukocyte cell-cell adhesion | 310 | 0 |
|  | GO:0002285 | lymphocyte activation involved in immune response | 167 | 0 |
|  | GO:0002764 | immune response-regulating signaling pathway | 452 | 0 |
|  | GO:0006959 | humoral immune response | 222 | 0 |
|  | GO:0002446 | neutrophil mediated immunity | 473 | 0 |
|  | GO:0001819 | positive regulation of cytokine production | 399 | 0 |
|  | GO:0002694 | regulation of leukocyte activation | 461 | 0 |
| **CC** | GO:0031983 | vesicle lumen | 320 | 0 |
|  | GO:0072562 | blood microparticle | 103 | 0 |
|  | GO:0030667 | secretory granule membrane | 280 | 0 |
|  | GO:0001772 | immunological synapse | 32 | 0 |
|  | GO:0042611 | MHC protein complex | 19 | 0 |
|  | GO:0098636 | protein complex involved in cell adhesion | 35 | 0 |
|  | GO:0031091 | platelet alpha granule | 88 | 0 |
|  | GO:0030055 | cell-substrate junction | 395 | 0 |
|  | GO:0030139 | endocytic vesicle | 281 | 0 |
|  | GO:0098552 | side of membrane | 460 | 0 |
| **MF** | GO:0003823 | antigen binding | 52 | 0 |
|  | GO:0004896 | cytokine receptor activity | 88 | 0 |
|  | GO:0019865 | immunoglobulin binding | 22 | 0 |
|  | GO:0019955 | cytokine binding | 119 | 0 |
|  | GO:0030594 | neurotransmitter receptor activity | 114 | 0 |
|  | GO:0042165 | neurotransmitter binding | 52 | 0 |
|  | GO:0061134 | peptidase regulator activity | 202 | 0 |
|  | GO:0035254 | glutamate receptor binding | 45 | 0 |
|  | GO:0005126 | cytokine receptor binding | 258 | 0 |
|  | GO:0042287 | MHC protein binding | 24 | 0.003 |
| **KEGG Pathway** | hsa04062 | Chemokine signaling pathway | 71 | 0 |
|  | hsa04672 | Intestinal immune network for IgA production | 45 | 0 |
|  | hsa04064 | NF-kappa B signaling pathway | 90 | 0 |
|  | hsa04658 | Th1 and Th2 cell differentiation | 90 | 0 |
|  | hsa04659 | Th17 cell differentiation | 105 | 0 |
|  | hsa04514 | Cell adhesion molecules (CAMs) | 137 | 0 |
|  | hsa04662 | B cell receptor signaling pathway | 70 | 0 |
|  | hsa04666 | Fc gamma R-mediated phagocytosis | 86 | 0 |
|  | hsa04650 | Natural killer cell mediated cytotoxicity | 123 | 0 |

Note: LGG, Brain Lower Grade Glioma; FCGR3A, Fc fragment of IgG receptor IIIa; GSEA, Gene Set Enrichment Analysis; BP, biological process; CC, cellular component; MF, molecular function; GO, Gene Ontology; KEGG, Kyoto Encyclopedia of Genes and Genomes.
